# The Influence of Different Preservation Protocols on the Teeth’s Osteoinductive Characteristics: An In Vitro Study

**DOI:** 10.3390/ijms26094044

**Published:** 2025-04-24

**Authors:** Filiberto Mastrangelo, Sara Franceschelli, Ciro Annicchiarico, Alice Annicchiarico, Maria Elena Bizzoca, Federica De Cecco, Rosalba La Gioia, Gabriele Cervino, Mirko Pesce

**Affiliations:** 1 Department of Clinical and Experimental Medicine, University of Foggia, Via Rovelli n. 60, 77100 Foggia, Italy; 2 Department of Medicine and Aging Sciences, University “G. d’Annunzio” Chieti-Pescara, Via dei Vestini 31, 66100 Chieti, Italy; 3 Independent Researcher, Viale Ferruccio Parri, 6, 74023 Grottaglie, Italy; 4 Dentistry Student, University “A. Moro” Bari Piazza Umberto I, 1, 70121 Bari, Italy; 5 Independent Researcher, 70121 Bari, Italy; 6 Department of Biomedical, Dental and Morphological and Functional Imaging Sciences, University of Messina Piazza Pugliatti, 98122 Messina, Italy

**Keywords:** Tooth Transformer, osteoinductivity, bone morphogenetic protein-2, mineralization protein LIM-1, transforming growth factor-β

## Abstract

The purpose of this study was to evaluate in vitro whether the type of tooth preservation before treatment with the Tooth Transformer^®^ (TT) device affects the osteoinductive characteristics of the extracted tooth. Forty extracted teeth from healthy non-smoking patients were selected. All teeth were cleaned of caries, tartar, and filling material and then roughly sectioned and divided into four experimental groups according to storage type: room-temperature (RT) tooth samples, frozen tooth samples, RIPA tooth samples, and fresh tooth samples. Each sample was minced, demineralized, and disinfected using the TT device. The Enzyme-Linked ImmunoSorbent Assay (ELISA) test revealed the presence of bone morphogenetic protein-2 (BMP-2) and collagen type-I (COL-I) in all of the samples, demonstrating that the fresh teeth retained the most significant amount of osteoinductive protein. In contrast, the tooth samples stored at room temperature (RT) showed the most important loss of BMP-2 and COL-I. A Western Blot analysis demonstrated the presence of the Mineralization Protein LIM-1 (LMP-1) and Transforming Growth Factor-β (TGF-β) in all of the dental samples analyzed. The fresh and frozen dental samples showed significantly higher levels of LMP-1 than those in the other samples. In contrast, the levels of TGF-β were similar in all of the dental samples examined, regardless of the type of storage. These experimental results suggest that an extracted tooth should be treated with the TT device as soon as possible to maximize its osteoinductive potential in surgical bone preservation and regeneration procedures.

## 1. Introduction

Partial or total edentulism of the jaw bones seriously impacts a patient’s social life and reduces their quality of life by affecting their aesthetic, masticatory, and phonatory functions. Unlike in the past, when many patients considered tooth loss almost inevitable, nowadays, most people perceive missing teeth, especially front teeth, as a very adverse event [1]. The psychological consequences of tooth loss are a lack of self-confidence, non-acceptance of the change in facial shape, limitation of food choices, decreased enjoyment of food, avoidance of laughing in public, and reluctance establishing close relationships [2].

Edentulism can cause a reduction in facial height, a decreased mouth width, flattening of the profile, and lip retrusion [3]. Studies have consistently shown that individuals with complete dentures have much lower masticatory efficiency than that in individuals with a natural dentition. This is partly due to the loss of nerve fibres and the central pattern generator in the brainstem that coordinates chewing movements [4]. As a result, edentulous patients often avoid hard-to-chew foods like fruits and vegetables, opting instead for softer, more processed foods. This dietary shift is linked to increased cardiovascular risk and a higher incidence of certain cancers and age-related changes, while higher fruit and vegetable intake is associated with a reduced risk of oesophageal, gastric, and colorectal cancers [5,6,7].

Tooth loss triggers adaptive changes in both the soft and hard tissues, leading to progressive resorption of the edentulous alveolar ridge. The alveolar bone, which shares a mesenchymal embryonic origin with the teeth, loses its functional masticatory load after tooth extraction, resulting in three-dimensional bone resorption—more pronounced on the buccal side than the lingual or palatal side [8].

In 2005, Araújo and Lindhe’s study revealed that after eight weeks of healing, the buccal bone wall in post-extraction sockets was 2.2 mm more apical, while the lingual wall was 0.1 mm more apical, indicating greater resorption at the buccal surface [9]. A systematic review showed a horizontal bone loss of 32% at 3 months and 29–63% at 6–7 months and a vertical bone loss of 11–22% at 6 months [10].

Recent studies have assessed several titanium and graft biomaterials for reconstructing bone defects [11]. The available options include autologous, homologous, heterologous, and synthetic materials [12].

Autologous bone grafts are the gold standard due to their biocompatibility and osteogenic properties, but they can lead to morbidity and rapid resorption. Allografts, xenografts, and alloplastic grafts, with and without mesenchymal stem cells, provide good osteoconductive potential but lack osteogenic and osteoinductive properties [13,14].

Allografts, xenografts, and alloplastic grafts offer good osteoconductive but not osteoinductive and osteogenetic potential [15].

As part of the search for a new bone grafting material to promote bone tissue regeneration, extracted teeth have been considered due to their similar chemical composition and embryological origin to those of alveolar bone [16]. Dentin consists of 70% inorganic material, 20% organic material, and 10% water. Alveolar bone, on the other hand, has 65% inorganic content, 25% organic content, and 10% water content [17]. The organic component of dentin is at least 90% collagen type-I (COL-I), which plays a key role in the formation and mineralization of the osteoid matrix. The remaining 10% of the organic component of dentin consists of Non-Collagenous Proteins (NCPs), such as bone morphogenetic proteins (BMPs), Transforming Growth Factor-β (TGF-β), and Mineralization Protein LIM-1 (LMP-1) [18].

BMPs promote the differentiation of the bone marrow mesenchymal stem cells into chondrocytes, facilitating bone tissue mineralization [19].

Transforming Growth Factor-β (TGF-β) regulates the proliferation and early differentiation of osteoprogenitor cells into osteoblasts through the selective MAPK and Smad 2/3 pathways and cooperation with PTH, Wnt, BMPs, and FGF [20].

Mineralization Protein LIM-1 (LMP-1) promotes osteoblast differentiation and is associated with bone morphogenetic protein-6 (BMP-6), indicating its role in the BMP signaling pathway [21].

The inorganic component of dentin consists of calcium and phosphate ions that form hydroxyapatite crystals, which are larger than those found in bone and much smaller than those that make up enamel [22].

The organic component of dentin gives the tooth osteoinductive potential, while the inorganic component grants it osteoconductive properties [23].

AutoBT, an autogenous bone graft material developed from an extracted tooth, has been used clinically in Korea since 2008 [18]. AutoBT consists of low-crystallinity hydroxyapatite (HA), tricalcium phosphate (TCP), amorphous calcium phosphate (ACP), and octacalcium phosphate (OCP), so it has osteoconductive potential. AutoBT also has osteoinductive potential; in fact, it contains BMP-2 and TGF-β [24].

Gupta et al. compared the effectiveness of AutoBT and mandibular autogenous bone grafting in preserving post-extraction sockets [25]. Radiographic examination showed that AutoBT sites had greater radiopacity due to its higher mineral content compared to that of autogenous bone grafts.

The Tooth Transformer^®^ (TT) is a medical device that allows autologous bone graft material to be obtained from an extracted tooth. It reduces the crystallinity of hydroxyapatite, making the morphogenetic proteins and growth factors present in dentin available [26].

Minetti et al. demonstrated that chemical demineralization treatment with the Tooth Transformer^®^ device makes dentin resemble bone [27].

An interesting case report involved a patient who underwent guided bone regeneration (GBR) surgery in the 2.6 atrophic area, using a 3.8 previously extracted from the same patient and treated at the time of GBR as the graft material, with the Tooth Transformer^®^ device [28]. After four months of healing, the radiographic investigation showed a bone gain in height of 9.82 mm, which allowed implant placement surgery to be planned.

Interesting experimental results obtained by our research group showed that chemical demineralization treatment with the Tooth Transformer^®^ device did not biologically alter the osteoinductivity of an extracted and dry-stored human tooth [29,30,31].

The present in vitro study aims to evaluate whether the osteoinductive characteristics of an extracted human tooth treated with the Tooth Transformer^®^ device are affected by the mode of tooth storage.

## 2. Results

### 2.1. A Comparative Analysis of the BMP-2 and COL-1 Expression in RT, Frozen, RIPA, and Fresh Tooth Samples

The Enzyme-Linked ImmunoSorbent Assay (ELISA) test was used to quantify the content of the osteoinductive proteins bone morphogenetic protein-2 (BMP-2) and collagen type-I (COL-I) in the four experimental groups of dental samples treated using the TT device.

**Figure 1:**
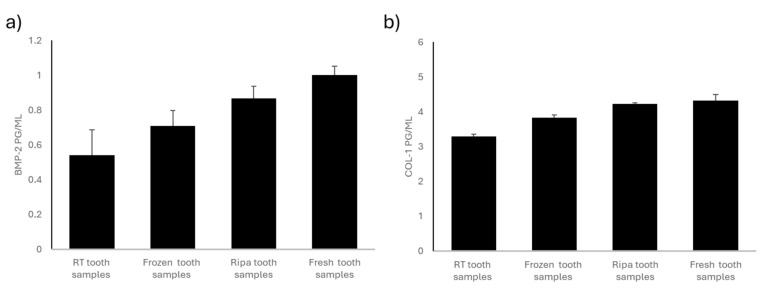
BMP-2 and COL-1 levels in the four experimental groups: room-temperature (RT) tooth samples, frozen tooth samples, RIPA tooth samples, and fresh tooth samples. (**a**) BMP-2 content (pg/mL). BMP-2 levels are highest in the fresh teeth and RIPA buffer specimens, followed by the frozen specimens, with the lowest levels found in the room-temperature samples. Data are expressed as standard error ± average (SE). (**b**) The COL-1 content is highest in the fresh teeth, followed by that in the specimens stored in RIPA buffer and those frozen, while the lowest levels are observed in the specimens kept at room temperature. Data are expressed as standard error ± average (SE).

The dental samples stored at room temperature (RT) and in a dry environment showed a significantly lower concentration of BMP-2 than that in the other experimental groups examined (Figure 1a). This loss of osteoinductive protein is likely due to the lack of an adequate mode of storage of the dental samples.

The dental samples stored by freezing them at −20 °C showed greater preservation of the osteoinductive BMP-2 protein content than that in the dental samples stored at room temperature (RT). However, these samples had a significantly lower BMP-2 protein content than that in the tooth samples stored in RIPA and the fresh tooth samples (Figure 1a). Tooth preservation through freezing at −20 °C would be effective in slowing down protein degradation. The partial loss of osteoinductive proteins may have been due to the freezing and thawing cycles.

The dental samples preserved through treatment with RIPA buffer solution and then frozen at −20 °C showed intermediate values for BMP-2 (Figure 1a). The RIPA buffer solution contains protease inhibitors; it is designed to preserve the protein structure by preventing its enzymatic degradation. The concentration of BMP-2 in the tooth samples stored in RIPA was lower than that in the fresh teeth but still higher than that in the teeth stored at room temperature (RT) and the frozen teeth.

The fresh tooth samples i.e., those processed using the Tooth Transformer^®^ device immediately after their collection, showed the highest BMP-2 content (Figure 1a). The proteins did not undergo degradation related to the storage mode, so these tooth samples retained their full osteoinductive potential. Immediate treatment ensured minimal loss of BMP-2, making this method the gold standard for preserving the osteoinductivity of dental elements. Compared to any other preservation method, the fresh tooth samples stained the most significant amount of BMP-2, suggesting the importance of treating extracted teeth as soon as possible.

The frozen, RIPA-preserved, and fresh tooth samples showed no significant changes in COL-I content, suggesting that these preservation methods did not significantly influence the collagen content of the tooth samples tested (Figure 1b). On the contrary, a significant reduction in COL-I content was found in the tooth samples stored at room temperature (RT) and in a dry environment compared to that in the other experimental groups (Figure 1b), emphasizing the need for an appropriate way to store teeth to prevent collagen degradation.

### 2.2. Evaluation of Mineralization Protein LIM-1 (LMP-1) and Transforming Growth Factor-β (TGF-β)

**Figure 2:**
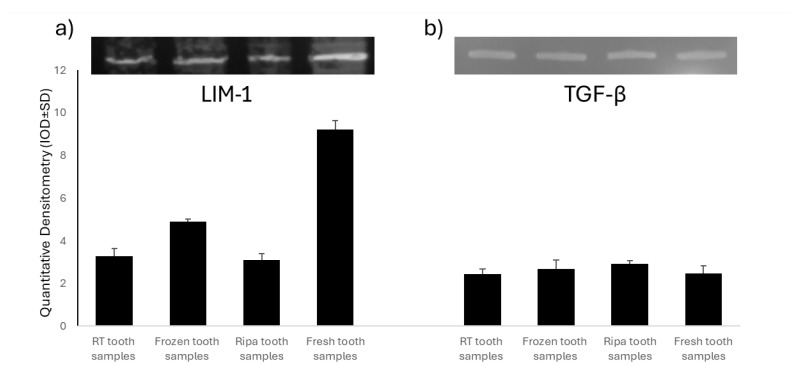
Analysis of Mineralization Protein LIM-1 (LMP-1) and Transforming Growth Factor-β (TGF-β) in the four experimental groups: room-temperature (RT) tooth samples, frozen tooth samples, RIPA tooth samples, and fresh tooth samples. Representative images of the Western blot analysis of LMP-1 (**a**) and TGF-β (**b**).

A Western Blot analysis was used to assess the expression of Mineralization Protein LIM-1 (LMP-1) and Transforming Growth Factor-β (TGF-β) in the four experimental groups of dental samples treated using the TT device. LMP-1 is a key intracellular regulator of osteogenesis, capable of increasing the expression of several osteoinductive factors, including BMP-2, and modulating TGF-β activity. The experimental results showed the presence of LMP-1 in all of the samples tested (Figure 2), with significantly higher levels in the fresh tooth samples and those frozen at −20 °C. The high expression of LMP-1 in the fresh tooth samples further supports the hypothesis that immediate treatment of an extracted tooth with the TT device optimally preserves key bioactive factors for bone regeneration. In contrast, the TGF-β levels (Figure 2) were similar in all of the samples examined, regardless of the tooth preservation protocol.

## 3. Discussion

In the current social context, oral health has taken on fundamental importance for interpersonal relationships, as well as for improving quality of life and self-perception [32].

The lack of mechanical function following the loss or extraction of dental elements leads to physiological vertical and horizontal resorption of the alveolar bone ridge [33].

The performance of surgical procedures aiming to regenerate bone tissue is therefore essential in order to ensure a volume of bone compatible with the safe and predictable rehabilitation of the edentulous ridge [34]. To date, the surgical techniques for hard tissue regeneration involve the use of the following biomaterials: autologous bone, homologous bone, heterologous or xenologous bone, and synthetic biomaterials [35].

Autologous bone, grafted in order to prevent or reduce the physiological bone resorption that follows tooth loss, is still considered the reference material today because it has documented osteogenic, osteoinductive, and osteoconductive characteristics. However, the use of this biomaterial is limited by the morbidity of the intra-oral or extra-oral donor site, as well as the limited availability of tissue that can be harvested and the rapid rate of resorption [36,37].

Homologous bone is bone graft material that is taken from human cadavers, subjected to freezing or demineralization treatment, and then sterilized and supplied in the form of particles or bone blocks by licensed tissue banks. The use of homologous bone is limited by its protein content, which exposes it to the risk of cross-infection and immunological reactions [38].

Xenografts consist of the use of a tissue derived from another animal species (bovine, porcine, or equine). This biomaterial is obtained using a chemical or physical (low-heat) process that preserves the original bone architecture and mineral component of the bone but removes the organic component in order to avoid immune reactions. Xenografts are characterized by biocompatibility, osteoconductive potential, and a very slow rate of resorption [39].

Synthetic biomaterials consist of different combinations of calcium phosphate, bioactive glasses, and polymers. Hydroxyapatite (HA) is the main mineral component of bone; it has osteoconductive characteristics and is highly resistant to physiological resorption. In contrast, beta-tricalcium phosphate (β-TCP) undergoes rapid resorption and replacement by the host tissue during the early stages of healing [40].

Considering the limitations of commercially available bone graft biomaterials, several research groups have examined the possibility of using extracted teeth, usually discarded as waste, as an autologous bone substitute in alveolar bone preservation and regeneration procedures [41]. The tooth and alveolar bone have the same embryonic mesenchymal origin and a similar chemical composition. The organic component of dentin and alveolar bone is 90% collagen type-I (COL-I), which plays a supporting role during the deposition and mineralization processes of the osteoid matrix. The remaining 10% of dentin’s organic content consists of non-collagenous proteins (NCPs), including bone morphogenetic proteins (BMPs) and Transforming Growth Factor-β (TGF-β) [42].

Bone morphogenetic proteins (BMPs) induce the differentiation of the mesenchymal stem cells from the bone marrow into osteoblasts, i.e., cells responsible for the production and deposition of the osteoid matrix, as well as playing a key role in promoting the process of mineralization of the matrix deposited to give bone tissue [43]. The direct injection of soluble BMPs into an affected site is not effective for bone regeneration due to their rapid diffusion, so organic or inorganic materials have been introduced to ensure their controlled delivery. Among these materials, hydroxyapatite (HA) is able to deliver BMP-2 for the treatment of large bone defects [44].

Transforming Growth Factor-β (TGF-β) is involved in the induction of mesenchymal stem cell aggregation and differentiation, as well as promoting bone tissue healing [45].

Transforming Growth Factor-β (TGF-β) and bone morphogenetic proteins (BMPs) belong to the TGF-β superfamily. Transmitting signals via SMAD-dependent and independent pathways, they are involved in the recruitment and differentiation of osteoblasts and chondrocytes, as well as playing an essential role in skeletal development and the maintenance of homeostasis. BMPs induce osteogenesis, osteoclastogenesis, and chondrogenesis at all stages of differentiation, while TGF-β has a different function depending on the stage. TGF-β is involved in the maintenance of the osteocyte network and has an opposite role to BMPs in the homeostasis of the articular cartilage. The signaling pathway of BMPs and TGF-β requires a multi-level regulatory mechanism, through control over latency in the matrix, extracellular antagonists, ubiquitination, and phosphorylation in the cytoplasm, nucleus–cytoplasmic transport, and transcriptional coregulation in the nuclei [20].

Kim et al. subjected extracted tooth to dehydration, degreasing, and disinfection treatments with ethylene oxide in order to produce a granulate of AutoBT (Korea Tooth Bank, Korea), a new type of autologous bone graft biomaterial [46]. The authors used AutoBT for guided bone regeneration (GBR) and the simultaneous placement of implants at the same site and found new bone formation in 46–87% of the area of interest during the first 3–6 months of healing.

In 2018, Peng Li et al. published a study whose aim was to evaluate the clinical efficacy of an autogenous demineralized dentin matrix (DDM) compared to that of Bio-Oss granules in a guided bone regeneration (GBR) surgical procedure aiming to place post-extractive implants [47]. No statistically significant difference was found with regard to the implant stability and marginal bone resorption, so the authors concluded that autologous DDM granule grafting could be an excellent alternative to Bio-Oss in GBR.

Gomes et al. performed biochemical, radiographic, and histological analyses to evaluate the effects of a demineralized dentin matrix (DDM) and Platelet-Rich Plasma (PRP) in the guided bone regeneration (GBR) treatment of surgical defects created in the parietal bones in diabetic rabbits [48]. The DDM was shown to have osteoinductive and osteoconductive potential; in fact, it induces chemotaxis of the osteoprogenitor cells and osteoblasts, while PRP is able to stimulate angiogenesis and red bone marrow formation.

A demineralized dentin matrix (DDM) can be produced using the Tooth Transformer^®^ device and two other commercially available devices, the BonMaker and the Smart Dentin Grinder. Block et al. [39] compared the clinical effectiveness of the demineralized dentin matrices obtained using these three devices in bone regeneration and implant placement procedures after three months [32]. All patients showed optimal bone regeneration and adequate osseointegration of the implant. Their SEM analysis showed the different particle sizes produced by the Smart Dentin Grinder (300–1300 μm), Tooth Transformer^®^ (30 μm-1.1 mm), and BonMaker (500–1000 μm).

In 2023, Francescelli et al. published a study on the use of extracted teeth treated using the Tooth Transformer^®^ device in the immediate implant placement surgical procedure [29]. At the six-month follow-up, the Pink Esthetic Score (PES) index showed an average score of 8, resulting in an aesthetically adequate result with respect to the condition of the peri-implant soft tissue. The mesial and distal bone resorption at the six-month follow-up was 0.39 ± 1.19 mm and 0.42 ± 0.90 mm, respectively. All of the patients were satisfied with their masticatory, phonatory, and aesthetic function.

A pilot study examined the possibility of using autologous demineralized dentine for a sinus lift surgical procedure [35]. An increase in the bone ridge height of 14.72 ± 2.83 mm and an implant survival rate of 97.5% after 20 months of follow-up were found.

The present in vitro study was conducted with the aim of assessing for the first time whether or not the osteoinductivity characteristics of extracted teeth treated using the Tooth Transformer^®^ device are influenced by the method of tooth preservation. The final objective was to identify the ideal method for preserving the extracted tooth so that it could be used as an ideal substitute for autologous bone, which is still the reference material in the field of oral bone regeneration. Our research group selected forty extracted teeth and divided them into four experimental groups, each consisting of ten tooth samples. Each experimental group represented a different method of dental preservation: specimens preserved at room temperature (RT) and in a dry environment, specimens preserved through freezing at −20 °C, specimens preserved in RIPA buffer solution and frozen at −20 °C, and specimens processed fresh without any preservation treatment. Subsequently, the dental elements were subjected to grinding and chemical demineralization using the Tooth Transformer^®^ device to analyze their content of osteoinductive factors.

The tooth samples stored at room temperature (RT) and in a dry environment showed the lowest concentration of bone morphogenetic protein-2 (BMP-2) and collagen type-I (COL-I). The tooth samples frozen at −20 °C had a higher BMP-2 content than that in the teeth stored at room temperature (RT) but lower than that in the teeth stored in RIPA and the fresh teeth. The tooth samples stored in RIPA buffer solution showed an intermediate concentration of BMP-2. In contrast, the fresh tooth samples had the highest values of BMP-2, strongly suggesting that extracted teeth should be treated as soon as possible using the Tooth Transformer^®^ device to maximize their osteoinductive potential. When teeth are frozen at −20 °C, the BMP-2 preservation is more beneficial, as the cold environment slows down the dual processes of enzymatic and microbial degradation. However, the significant decrease in BMP-2 levels compared to those in the freshly extracted teeth suggests that even freezing, although beneficial, cannot completely stop the degradation of these valuable proteins. This persistent decline may be due to freeze–thaw cycles, which can cause gradual but damaging protein denaturation over time. Preserving the teeth in RIPA buffer produced intermediate levels of BMP-2, suggesting that protease inhibitors play a key role in limiting enzymatic degradation. However, this protective method is insufficient, as the BMP-2 levels in the RIPA-preserved teeth remained below those in the fresh samples. This finding is crucial for researchers and clinicians considering RIPA buffer for preservation, as it strikes a delicate balance between the convenience of conservation and the need to maintain protein stability. The experimental results also showed that the frozen, RIPA-preserved, and fresh tooth samples did not show significant changes in their collagen type-I content. Most dental preservation methods maintain COL-1 levels, highlighting its stability as a key structural protein. However, the apparent reduction in COL-1 levels in the samples stored at room temperature underscores the critical importance of using the optimal storage methods to prevent collagen degradation. In sharp contrast to COL-1, BMP-2 is known to have an intrinsically unstable biological nature. BMP-2 has been shown to be extremely sensitive to various methods of dental preservation and is particularly susceptible to degradation under adverse environmental conditions. Despite the need for further studies, the present in vitro analysis regarding the ideal storage method for extracted teeth would discourage room-temperature (RT) and dry storage methods in favor of immediate treatment of the fresh tooth elements without any additional storage methods.

The presence of Mineralization Protein LIM-1 (LMP-1) and Transforming Growth Factor-β (TGF-β) was also detected in all of the dental samples examined. The fresh tooth samples and the tooth samples frozen at −20 °C showed the highest levels of LMP-1, indicating the extreme sensitivity of this protein to environmental influences. In contrast, the TGF-β levels were similar in all of the samples tested regardless of the storage protocol used, underscoring the significantly greater stability of this protein compared to that of LMP-1. LMP-1 is an intracellular positive regulator essential for osteoblastic differentiation. Its temporal and spatial association with bone morphogenetic protein-6 (BMP-6) suggests the possible involvement of LMP-1 in the BMP signaling pathway, a family of proteins essential to bone formation. LMP-1 appears to be involved in both mechanisms of bone formation. BMP-6 and LMP-1 are essential to both the initiation of membranous bone formation and the final stage of endochondral ossification [49]. Pan et al. reported that LMP-1 regulates BMP-2 at the transcriptional level through modulation of Runt-related transcription factor 2 (Runx2) [50]. Runx2 is a member of the Runt domain family and is essential in osteoblastic differentiation and maturation. The activation of Runx2 in the osteoprogenitor cells during osteoblast differentiation and maturation results in the expression of osteoblastic markers such as collagen type-I (COL-I) and osteocalcin (OCN). The overexpression of Runx2 significantly increases the activity of the BMP-2 promoter with LMP-1. From a molecular point of view, ERK1/2 MAPK activation is crucial to the LMP-1-induced upregulation of the transactivity of Runx2 transcription factor and the subsequent induction of BMP-2. This crosstalk between BMP-2 and Runx2, activated by LMP-1, plays a vital role during osteoblastic differentiation and mineralization of the osteoid matrix produced [51].

## 4. Materials and Methods

### 4.1. Sample Preparation

Forty extracted teeth from healthy non-smoking patients were selected. None of the teeth had been endodontically treated and were extracted after obtaining informed signed consent from the 32 different patients (average age: 38 yrs; M-25, F-7). The 32 patients were randomly assigned into each group.

All of the procedures used were reported in n°638 Ethical Committee surgical protocol of the University of Chieti and approved. 

**Figure 3:**
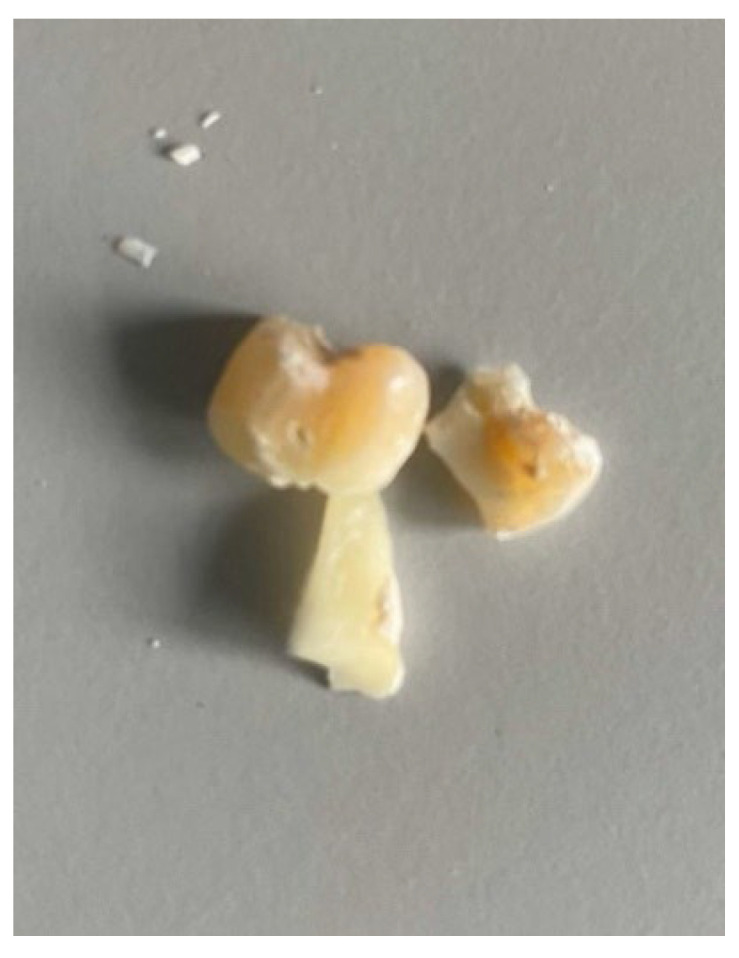
A cleaned and roughly sectioned tooth element.

Using truncated cone diamond burs on a turbine and under constant irrigation with chilled sterile saline, all of the teeth were cleaned of caries, tartar, and filling materials and then roughly sectioned (Figure 3).

The weight of the samples varied between 0.181 g and 1.396 g.

Depending on the mode of storage before treatment with the Tooth Transformer^®^ device (TT Tooth Transformer srl, Milan, Italy), the selected teeth were divided into four experimental groups.

All of the patients were of Italian nationality and of a good socioeconomic status. In order to obtain a homogeneous patient sample and to avoid potential confounding factors, only patients without relevant diseases and without chronic medication were included in this study.

1. Room-temperature (RT) tooth samples: Ten samples were stored at room temperature and in a dry environment. This experimental group included 10 patients, each of whom provided one tooth specimen (Table 1).

2. Frozen tooth samples: Ten samples were frozen at −20 °C immediately after collection without any additional treatment. This experimental group included 10 patients, each of whom provided one tooth sample (Table 2).

3. RIPA tooth samples: Ten samples were treated with a RIPA buffer solution (50 mM Tris-HCl, pH 7.4, 150 mM NaCl, 1% Triton X-100, 0.5% sodium deoxycholate, 0.1% SDS) for 30 min at 4 °C to achieve cell lysis and then frozen at −20 °C. This experimental group included 5 patients, 4 of whom provided one tooth sample each and one who underwent oral cavity remediation and provided 6 tooth samples (Table 3).

4. Fresh tooth samples: Ten samples were processed immediately after collection without any storage treatment. This experimental group included 7 patients, 5 of whom each provided one tooth sample, 1 of whom provided three tooth samples, and 1 of whom provided two tooth samples (Table 4).

**Figure 4:**
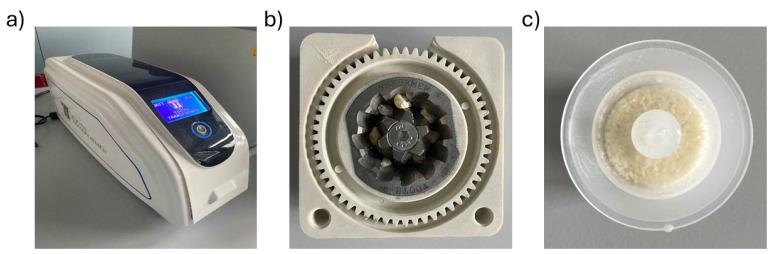
Components of the device. (**a**) Tooth Transformer^®^. (**b**) Dental fragments placed in the tooth grinder. (**c**) Granules were obtained at the end of treatment with the Tooth Transformer^®^ device.

Using the Tooth Transformer^®^ device (TT Tooth Transformer srl Milan, Italy) and following the manufacturer’s instructions, each sample was minced, demineralized, and disinfected (Figure 4a). Ethical committee approval was unnecessary, as the Tooth Transformer^®^ device is commercially available. Precisely, the tooth fragments were placed in the Tooth Grinder, i.e., a container made of thermoplastic material and equipped with surgical steel blades working at very low revolutions, so that they were ground to yield granules with a diameter ∅ < 1 mm (Figure 4b). The resulting particulate was demineralized, disinfected, and rinsed using a disposable accessory kit. The granules were exposed to UVA radiation and ultrasonic vibrations, with temperature variations always kept consistently below 43 °C to avoid damaging the proteins. At the end of the treatment (25 min), one basket contained the granules, while a cylinder contained the spent liquids. Subsequent analyses were performed on the granules (Figure 4c).

### 4.2. Reagents

TT Tooth Transformer srl (Milan, Italy) supplied the demineralization reagents. The demineralization kit contained a cartridge with six liquids: two hydrochloric acid (0.1 M HCl) and hydrogen peroxide (10% H_2_O_2_) samples, one demineralized water (H_2_O) sample, and four mineralized water (H_2_O) samples (Figure 2). These liquids were needed to remove the acidic residues in four different phases.

### 4.3. Measurement of BMP-2 and Collagen Type-I

The tissue levels of human bone morphogenetic protein-2 (BMP-2, CSB-E04507h) and human collagen type-I (CSB-E13445h) were measured using a commercial ELISA kit (Cusabio (Houston, TX, USA) according to the instructions of the productor. The plates were scanned using a cooled, specialized Charge-Coupled Device. The integrated density values of the spots of known standards were used to generate a standard curve. The density values for unknown samples were determined using the standard curve for each analysis to calculate the actual values in pg/mL. All steps were performed twice. The assay’s sensitivity was equal to 15.6 pg/mL. The intra- and inter-assay reproducibility was >90%. Duplicate values that differed from the mean by more than 10% were considered erroneous and therefore were repeated.

### 4.4. Western Blot Analysis

Following the treatment with the TT, ≈50 mg of the particles was transferred into 1.5 mL polypropylene tubes, and RIPA buffer was added to each sample. After incubation at 4 °C overnight and three freezing/thawing cycles, the samples were sonicated using a tip ultrasonicator, and protein was extracted. Total protein content was determined using a BCA protein assay kit, according to the manufacturer’s instructions (Thermofisher, Monza, Italy). The Western blot analysis was performed as described previously [26], using the following antibodies against Monoclonal Mouse LIM1 (R&D Systems, MAB2725; 1:1000): goat polyclonal TGF-β1 (R&D Systems, AF-246-NA; 1:300). The blots were then incubated for one hour at room temperature using a goat anti-mouse secondary antibody (Sc-2005; 1:2000; Santa Cruz Biotechnology) or a polyclonal goat anti-rabbit secondary antibody (Sc-66931; 1:5000; Santa Cruz Biotechnology). Nitrocellulose was scanned using a computerized densitometric system (Bio-Rad Gel Doc 1000, Milan, Italy). The protein levels were normalized to the protein content.

### 4.5. Statistical Analysis

The data obtained were analyzed using appropriate statistical tests (an ANOVA) to compare the different conservation methods and their impact on the biochemical parameters of the teeth.

## 5. Conclusions

More research is necessary to validate the interesting experimental findings from this study. This in vitro study allowed our research group to demonstrate for the first time that the osteoinductive properties of extracted teeth are influenced by the method of tooth preservation prior to grinding and chemical demineralization treatment using the Tooth Transformer^®^ device.

All of the tooth samples examined showed the presence of BMP-2, COL-I, LMP-1, and TGF-β, indicating that treatment with the Tooth Transformer^®^ does not alter the extracellular matrix (ECM) of the tooth. However, the experimental data revealed significant differences in the amounts of osteoinductive proteins present in the tested samples, suggesting that these amounts depended on the different storage methods used. Notably, storing the teeth at room temperature (RT) in a dry environment resulted in a substantial loss of osteoinductive proteins. In contrast, storage through freezing, using RIPA buffer solution, or maintaining fresh storage led to better preservation of the samples’ osteoinductive properties.

The observation of higher protein levels in the fresh tooth samples led us to conclude that extracted teeth should be treated using the Tooth Transformer^®^ device as soon as possible to maximize their osteoinductive potential. This experimental evidence could have significant clinical implications, highlighting the potential to use freshly extracted teeth as a viable alternative to the currently available bone substitutes. In fact, extracted teeth, which are often discarded as waste, have properties such as biocompatibility, non-immunogenicity, osteogenicity, osteoconductivity, and osteoinductivity, strongly suggesting their use in surgical procedures for bone preservation and regeneration.

## Figures and Tables

**Table 1:** RT tooth samples.

RT Tooth Samples	Tooth	Extraction Date	TT Treatment Date	Sex and Age of the Patient	Extraction Indication	Sample Weight (g)
1	3.1	14 January 2022	15 December 2022	M 37 y/o	Fracture	0.498
2	2.4	14 January 2022	15 December 2022	F 64 y/o	Periodontitis	0.689
3	3.7	14 January 2022	15 December 2022	F 58 y/o	Periodontitis	1.396
4	4.6	7 February 2022	15 December 2022	M 47 y/o	Caries	0.730
5	2.6	7 February 2022	15 December 2022	F 65 y/o	Caries	1.324
6	1.7	16 February 2022	16 December 2022	F 67 y/o	Periodontitis	1.051
7	2.6	16 February 2022	16 December 2022	M 34 y/o	Caries	0.376
8	1.5	16 February 2022	16 December 2022	M 39 y/o	Fracture	1.117
9	2.5	3 March 2022	16 December 2022	F 73 y/o	Periodontitis	0.181
10	3.8	3 March 2022	16 December 2022	M 24 y/o	Pericoronitis	1.387

**Table 2:** Frozen tooth samples.

Frozen Tooth Samples	Tooth	Extraction Date	TT Treatment Date	Sex and Age of the Patient	Extraction Indication	Sample Weight (g)
11	4.5	15 November 2023	23 January 2024	M 55 y/o	Caries	0.728
12	3.4	15 November 2023	23 January 2024	F 65 y/o	Caries	0.380
13	1.5	23 November 2023	23 January 2024	M 67 y/o	Periodontitis	0.910
14	3.8	5 December 2023	23 January 2024	F 24 y/o	Pericoronitis	0.831
15	3.8	11 December 2023	23 January 2024	F 54 y/o	Caries	0.378
16	4.8	15 December 2023	23 January 2024	M 22 y/o	Pericoronitis	0.689
17	1.1	18 December 2023	23 January 2024	M 72 y/o	Periodontitis	0.460
18	2.2	19 December 2023	23 January 2024	F 66 y/o	Periodontitis	0.391
19	3.5	3 May 2024	16 May 2024	M 51 y/o	Periodontitis	0.470
20	4.4	3 May 2024	16 May 2024	M 61 y/o	Periodontitis	0.520

**Table 3:** RIPA tooth samples.

RIPA Tooth Samples	Tooth	Extraction Date	TT Treatment Date	Sex and Age of the Patient	Extraction Indication	Sample Weight (g)
21	1.6	3 November 2023	06 November 2023	M 62 y/o	Caries	0.657
22	3.2	17 November 2023	20 November 2023	F 73 y/o	Remediation	0.393
23	4.1	17 November 2023	20 November 2023	F 73 y/o	Remediation	0.547
24	4.2	17 November 2023	20 November 2023	F 73 y/o	Remediation	0.438
25	3.1	17 November 2023	20 November 2023	F 73 y/o	Remediation	0.491
26	3.5	17 November 2023	20 November 2023	F 73 y/o	Remediation	0.492
27	4.3	17 November 2023	20 November 2023	F 73 y/o	Remediation	0.318
28	1.8	17 November 2023	20 November 2023	M 75 y/o	Periodontitis	1.272
29	3.8	16 May 2024	20 May 2024	M 25 y/o	Pericoronitis	0.440
30	1.2	16 May 2024	20 May 2024	M 66 y/o	Periodontitis	0.820

**Table 4:** Fresh tooth samples.

Fresh Tooth Samples	Tooth	Extraction Date	TT Treatment Date	Sex and Age of the Patient	Extraction Indication	Sample Weight (g)
31	2.5	16 May 2024	16 May 2024	F 68 y/o	Periodontitis	0.860
32	2.4	12 July 2024	12 July 2024	M 71 y/o	Remediation	0.450
33	2.5	12 July 2024	12 July 2024	M 71 y/o	Remediation	0.550
34	2.6	12 July 2024	12 July 2024	M 71 y/o	Remediation	0.290
35	1.6	12 July 2024	12 July 2024	F 66 y/o	Caries	0.550
36	3.5	12 July 2024	12 July 2024	M 61 y/o	Remediation	0.270
37	3.6	12 July 2024	12 July 2024	M 61 y/o	Remediation	0.320
38	2.5	12 July 2024	12 July 2024	F 57 y/o	Periodontitis	1.034
39	2.2	12 July 2024	12 July 2024	M 17 y/o	Fracture	0.763
40	4.4	12 July 2024	12 July 2024	M 74 y/o	Periodontitis	0.485

## Data Availability

Data contained within the article.
